# Mercury release of amalgams with various silver contents after exposure to bleaching agent

**DOI:** 10.15171/joddd.2016.019

**Published:** 2016-06-15

**Authors:** Mahmoud Bahari, Parnian Alizadeh Oskoee, Siavash Savadi Oskoee, Firoz Pouralibaba, Ali Morsali Ahari

**Affiliations:** ^1^Dental and Periodontal Research Center, Tabriz University of Medical Sciences, Tabriz, Iran; ^2^Assistant Professor, Department of Operative Dentistry, Dental Faculty, Tabriz University of Medical Sciences, Tabriz, Iran; ^3^Professor, Department of Operative Dentistry, Dental Faculty, Tabriz University of Medical Sciences, Tabriz, Iran; ^4^Assistant Professor, Department of Oral Medicine, Tabriz University of Medical Sciences, Tabriz, Iran; ^5^Assistant Professor, Department of Endodontics, Alborz University of Medical Sciences, Karaj, Iran

**Keywords:** Carbamide peroxide, mercury, silver mercury amalgam, tooth bleaching

## Abstract

***Background.*** Since it is possible for carbamide peroxide (CP) bleaching agent to contact old amalgam restorations, the present in vitro study evaluated the amount of dissolved mercury released from amalgam restorations with various percent-ages of silver content subsequent to the use of 15% CP.

***Methods.*** Thirty ANA 2000 amalgam disks with 43.1% silver content and thirty ANA 70 amalgam disks with 69.3% silver content were prepared. In each group, 15 samples were randomly placed in glass tubes containing 15% CP (as experimental groups) and the remaining 15 samples were placed in buffered phosphate solution (as control groups) with the same 3-mL volume for 48 hours. Subsequently, the amount of mercury dissolved in each test tube was measured using Mercury Analyzing System (Cold Vapor Atomic Absorption, MASLO, Shimadzu, Japan). Data was analyzed with two-way ANOVA and a post hoc Tukey test. (α = 0.05).

***Results.*** The amount of mercury released after exposure to CP was significantly higher than that released after exposure to buffered phosphate (P < 0.001). In addition, the amount of mercury released from dental amalgam with a silver content of 43% was significantly higher than that released from dental amalgam with a silver content of 69% (P < 0.001).

***Conclusion.*** The amount of mercury release is inversely proportional to the silver content of dental amalgam.

## Introduction

Mouthguard bleaching (MGB) with carbamide peroxide (CP) gel is effectively used for the treatment of discolored teeth.^1^ Ten percent CP breaks into 3.6% hydrogen peroxide (H_2_O_2_) and 6.4% urea; hydrogen peroxide is the most common active ingredient of bleaching agents.^2^

Widespread use of bleaching agents has raised concerns about its oxidative effects on soft tissues, tooth structures and restorations.^3^ However, most MGB products are considered relatively safe materials in relation to their systemic effects^1^ and their influence on dental hard tissues.^4^Apart from these aspects, another possible interaction is the one between peroxide and dental materials, the effect of which appears to be material-dependent. Several studies have evaluated the effect of bleaching agents on dental materials, such as glass-ionomer cements, ceramics and gold. The results of these studies have failed to show any significant effect of bleaching agents to induce major changes in these materials.^5-7^ However, the results of several in vitro studies have shown an increase in the release of mercury when amalgam is exposed to bleaching agents.^8-12^

Although bleaching gels are commonly applied to anterior teeth, excess bleaching materials might inadvertently come into contact with amalgam restorations on premolars and molars, and may increase the susceptibility of amalgam to corrosion and degradation.^11^ Bleaching agents, including CP, break into free radicals which can theoretically corrode metallic alloys such as amalgam in the proximity or on the teeth undergoing bleaching procedures to release mercury.^11^ Furthermore, some studies reported greening of tooth-amalgam interface, discoloration and perforation of bleaching tray during bleaching procedures.^13,14^

Mercury released from dental amalgam during MGB might undergo absorption by the oral mucosa and the respiratory and digestive systems, increasing total body mercury burden, which results in an increase in the risk of various systemic toxic effects.^15-17^Toxic mechanism of mercury is broad. It binds sulfur, which is present in structural and functional cellular proteins.^18-20^ Berlin et al^19^ demonstrated that mercury is capable of blocking the sulfhydryl active sites in enzymes, receptors, molecules involved in signaling, and transport channels of membranes. These mechanisms interrupt strategic cellular processes in different ways depending on genetic and micronutrient status factors.^19^ The effects brought about consist of a change in membrane permeability, an increase in oxidative stress, peroxidation of lipid membranes, disruption of mitochondrial function and changes in the synthesis of neurotransmitters, cytokines and hormones.^18,19^ These can result in variable and nonspecific symptoms which might not be detected until much damage is inflicted.^21^ Therefore, it is important that we find ways to decrease the amount of mercury released from amalgam restorations subsequent to home bleaching procedures.

The duration of bleaching treatment, pH and concentration of bleaching agents, aging processes and surface polish of amalgam restorations are factors that affect the release of mercury from amalgam restorations.^1,22^ It seems that these factors should be evaluated. Considering lack of sufficient research on the effect of alloy elemental content of dental amalgam on the amount of mercury released following exposure to bleaching agents, the present in vitro study evaluated the amount of mercury released from dental amalgam products available on the market with various silver contents (43% and 69%) following exposure to CP.

## Methods

This was an in vitro study which did not involve the use of any animals or human data or tissues, and thus, an ethics approval was not required.

ANA encapsulated dental amalgam (Nordiska Dental, Angelhom, Sweden) with 43.1% (ANA 2000) and 69.3% (ANA70) silver contents, which are available on the market, were selected for the purpose of the present study. Table 1 summarizes general composition details of ANA2000 and ANA70. Thirty ANA 2000 and thirty ANA 70 amalgam samples were prepared by the use of silicon molds measuring 5 × 10 × 3 mm. Mixing was carried out in an amalgamator (Dentomat 2, Degussa AG, Frankfurt, Germany) with the equipment adjustments set by the manufacturer (4000 rpm, 10 seconds for 43.1% silver content and 8 seconds for 69.3% silver content) according to manufacturer’s instructions. The amalgam mix was immediately condensed by hand in the silicon molds using similar standard hand condensers. All the procedures were performed by one expert operator. The samples were left in the molds for 60 minutes for the initial setting reaction to occur and then retrieved and placed in normal saline solution for 24 hours. Fifteen samples from each group were randomly placed in 15% CP (pH = 6.5) (43CP and 69CP as experimental groups) and the remaining 15 samples were placed in buffered phosphate (BP) solution (pH = 6.5) (43BP and 69BP as control groups) with the same solution level and a solution volume of 3 mL. The test tubes were incubated in a dry environment at 37ºC for 48 hours. Carbamide peroxide solution was prepared by dissolving 107.153 grams of its powder (Merck, Hamburg, Germany) in 0.1 Mol of buffered phosphate using Rotstein method.^11^

**Table 1:** General composition details of ANA amalgams

Amalgam	General Composition	Batch Number	Manufacturer
ANA 2000	Ag 43.1%, Sn 30.8%, Cu 26.1%	109-19	Nordiska Dental, Angelhom, Sweden
ANA 70	Ag 69.3%, Sn 19.4%, Cu 10.9%, Zn 0.4%	130-19	Nordiska Dental, Angelhom, Sweden

Then the samples were retrieved from the test tubes and the amount of dissolved mercury was measured by Mercury Analyzer System (MASLO, Shimadzu, Japan). The chemical reaction of the Mercury Analyzer System depends on the cold vapor atomic Absorption method. Briefly, Nitric acid 0.5% and sulfuric acid 10% were added to the solution tested in the presence of potassium permanganate 0.5% and potassium persulfate 0.5% so that the mercury present in the solution would convert into mercuric ions (Hg^++^) by oxidation. Any excess oxidative agent was neutralized with hydroxylamine hydrochloride (30 g of hydroxylamine hydrochloride in distilled water to 1 liter). Then stannous chloride (50 g stannous sulfate Added to 500 mL of 2 N sulfuric acid) was added to the solution to reduce mercury ions in the form of metallic mercury. An internal pump was used to circulate air in a closed loop system through the solution that evaporated mercury and carried it through the absorption cell. The mercury vapor, which is in atomic form, is capable of absorbing light at a wavelength of 253.7 nm. A UV-sensitive phototube detects changes in the energy transmitted through the cell. The mercury concentration (μg/mL) in each test sample was determined using a standard curve generated by known concentrations of mercury.^11,23^

Different superscript symbol means statistically significant differences.

### Statistical analysis 

Subsequent to evaluating the normal distribution of data with Kolmogorov-Smirnov test and the equality of variances between the groups with Levene’s test, Data was analyzed with 2-way ANOVA and a post hoe Tukey test was used for the two-by-two comparison of the groups. Statistical significance was defined at P < 0.05.

## Results

Table 2 shows the means and standard deviations of the amount of mercury (μg/mL) released in each group. The results of two-way ANOVA showed the significant effect of type of solution and silver content of amalgam on the amount of mercury released (P < 0.001). In addition, the cumulative effect of these two factors was significant (P < 0.001).

**Table 2:** Means and standard deviations of the amount of mercury released in the experimental groups

**Solution**	**Silver (Ag) content of amalgam**	**Hg (μg/mL)** **Mean (SD)**
**Buffered phosphate**	43%	23.1 (2.92)^*^
	69%	21.70 (0.9)^*^
**Carbamide peroxide**	43%	49.26 (13.04)^*^
	69%	26.5 (7.46)^*^

Different superscript symbol means statistically significant differences.

Post hoc Tukey tests showed that the amount of mercury released under the influence of carbamide peroxide was significantly higher than that under the influence of buffered phosphate (P < 0.001). In addition, the amount of mercury released from dental amalgam with a silver content of 43% was significantly higher than that released from dental amalgam with a silver content of 69% (P < 0.001). Regarding the significance of the cumulative effect of the two variables, two-by-two comparison of the groups with a post hoc Tukey test showed significant differences between 43BP and 43CP groups (P < 0.001), 69BP and 43CP groups (P < 0.001), and 43CP and 69CP groups (P < 0.001). However, the differences between 43BP and 69CP groups (P = 0.92), 69BP and 69CP groups (P = 0.31), and 43BP and 69BP groups (P = 0.18) were not statistically significant.

## Discussion

Dental amalgam is a complex biomaterial, which is composed of 8-10 different phases, each with its specific microstructure. The corrosion behavior and dissolution of such a structure depends on the characteristics of each individual phase and on the electrochemical interaction between these phases in a special environment such as the oral cavity during MGB.^22,24^

In the present study the amount of mercury released from dental amalgams with silver content of 43% and 69% was compared after 48 hours of exposure to carbamide peroxide and BP. Although 48 hours of continuous exposure to CP is different than the usual clinical application duration in an intermittent manner, the results of the present study are important from a clinical point of view. Because CP is usually repeatedly applied during MGB and mercury release from amalgam is related to an increase in amalgam surface oxidation, corrosion and dissolution, which occur during the frequent and intermittent exposure to the products of CP disintegration.^11^The protocol used coincide with the protocols of studies in which bleaching agents have been applied continuously for several days in order to simulate the cumulative effect of bleaching agents during a period of time.^25,26^

In the present study the commercially available powder of carbamide peroxide was used, which is produced under pH controlled conditions; this method yields more reliable results compared to situations in which commercial gels are used. Not all commercially available tooth bleaching gels have the same chemical composition, viscosity and disintegration products. It has been demonstrated that different commercially available bleaching gels exert different effects on admixed dental amalgams.^10^

The results of this study showed that mercury is released from dental amalgam under physiologic conditions; too; however, its release significantly increases after exposure to CP, which is consistent with the results of previous studies.^8-12^ Carbamide peroxide breaks into hydrogen peroxide and urea with a proportion of 1/3 to 2/3. Hydrogen peroxide is the active ingredients of bleaching agents, including that of CP. The effect of bleaching agents on mercury release from dental amalgam is attributed to oxidation and reduction potential of hydroxyl ions.^1^ Free perhydroxyl radicals have a high oxidative activity and can exert an effect on microstructures containing silver-mercury.^1,27^ The silver-mercury phase (γ_1_) is the matrix of the dental amalgam structure and has a strong effect on its mechanical behavior and interaction with the environment. In addition, it is the principal source of mercury released from amalgam restorations. As a part of the dental amalgam structure, γ_1_ contains about 67-70% Hg.^28^

The first step in the release of mercury from dental amalgam is the dissolution of mercury due to the usually wet surface of amalgam restorations in the oral cavity.^29^ Since CP can interact with certain amalgam phases and affect the physicochemical behavior of amalgam restoration,^22^ it might facilitate the dissolution of γ_1_ phase and release of mercury into the solution. This facilitation is probably mediated through elimination of surface protective films, which facilities the degradation of amalgam surface and exposure of silver-mercury (γ_1_) phase. Based on the results of previous study, the amount of mercury released from γ_1_ phase is significantly higher than that of released from Ag-Hg-Sn phase and the dental amalgam itself.^30^ One of the most important protective factors is the presence of surface oxides, especially tin oxide. Tin deactivates γ_1_ phase and reduces solubility and release of mercury.^28,31^

Another interesting finding of the present study was that the amount of dissolved mercury released from dental amalgam with a silver content of 43% (tin content 30.8%, copper content 26.1%) was higher than that released from dental amalgam with a silver content of 69% (tin content 19.4%, copper content 10.9%). There are several possible factors which may contribute to this. One may be the lower concentration of tin in the γ_1_ phase of ANA2000 as compared with the ANA70, because it has been suggested that it plays a role in stabilizing the structure of γ_1_.^31^ Another factor may be that the unreacted particles in ANA70 provide a more efficient “sink” for mercury than do the particles in ANA2000, due to a higher concentration of silver in the former.^31^This, in part, might be attributed to the fact that Hg is sucked away from the amalgam surface and into its bulk.^32^ Therefore, amalgamation in the bulk might continue, despite the fact that the reaction at the surface has stopped as a result of “Hg starvation”.^31^ This may be due to the fact that, the rate of dissolution of silver in mercury is higher than that of tin.^33^ In contrast, mercury does not appear to “wet” the low silver-high copper alloy powder surface evenly. It is believed that mercury formed as droplets on the surface without spreading, and the reaction occurred locally under the droplets.^34^ The third contributing factor may be related to the fact that ANA70 in this study contained a small amount of zinc; it is possible to attribute any specific influence on mercury dissolution to zinc. However, one might speculate that as a result of the additional oxide, dissolution might be less in these amalgams compared to those without zinc under similar conditions.^35^

Regarding limitations for this in vitro study, it should be pointed out that caution should be exercised in extending the results of the present in vitro study to clinical situations because this study was performed on freshly mixed amalgam, Furthermore, it was not possible to simulate the rinsing and cleaning effect of the saliva, the effect of biofilm and the effect of pH changes in the oral cavity on the surface oxide layer. Considering the importance of the issue, it should be noted that saliva is a good electrolyte and might contribute to the release of mercury from amalgam due to its role in galvanic currents. Amalgam fillings, in contact with neighborhood amalgam, may also produce electrical currents that accelerate the release of mercury.^36^

Commercially available dental amalgams have other alloy contents in addition to Ag and Sn, and some impurities which might influence mercury stability in γ_1_ phase, and the composition, structure and surface oxide properties. Therefore, it is suggested that the tin oxide content, various phases and tin content of γ_1_ phase on the surface of amalgams with various silver contents be evaluated in future studies.

## Conclusions

The amount of mercury release is inversely proportional to the silver content of dental amalgam.

## Acknowledgments

The authors thank Dr. Majid Abdolrahimi for editing the English manuscript.

## Authors’ contributions

MB was responsible for the concept design and hypothesis formulation. Experimental design was performed by MB and PAO. AMA performed the experiments. SSO and FP were responsible for statistical analysis, and statistical interpretation of the findings. MB drafted the manuscript. All authors critically revised the manuscript for intellectual content. All authors have read and approved the final manuscript.

## Funding

This project was carried out by the financial support from the Deputy Dean of Research at Tabriz University of Medical Sciences.

## Competing interests

Dr. Siavash Savadi Oskoee is an Editorial Board member of the *Journal of Dental Research, Dental Clinics, Dental Prospects*. The authors have no other competing interests with regards to authorship and/or publications of this paper.

## Ethics approval

Not applicable.
